# Exceptional Responses to Standard Therapy in a Patient with Metastatic HER2-Positive Breast Cancer

**DOI:** 10.7759/cureus.1412

**Published:** 2017-06-29

**Authors:** Carsten Nieder, Bård Mannsåker, Ellinor Haukland

**Affiliations:** 1 Dept. of Oncology and Palliative Medicine, Nordland Hospital Trust

**Keywords:** breast cancer, brain metastases, radiotherapy, trastuzumab, chemotherapy, her2 positive breast cancer

## Abstract

Patients with metastatic breast cancer involving the liver and brain often have short overall survival. Here, we report a case of de novo metastatic breast cancer with multiple liver metastases at initial diagnosis in February 2011 in a 35-year-old Caucasian female patient. The histology was poorly differentiated invasive ductal carcinoma (estrogen and progesterone receptor negative, HER2 positive) and the patient was negative for germline BRCA 1 and 2 mutations. Systemic therapy with trastuzumab and docetaxel was given for six months and then switched to trastuzumab only because of peripheral neuropathy. At that time, the patient was in complete clinical remission. She developed brain metastases in September 2012 and received whole-brain radiotherapy, which resulted in complete remission. While on continued trastuzumab, the primary tumor in the breast recurred in May 2016. A mastectomy was performed and afterwards systemic therapy was intensified (trastuzumab, pertuzumab, paclitaxel). At the last follow-up (March 06, 2017) no further recurrence was detected. This case illustrates that standard HER2-directed treatment might provide long-term disease control also in selected patients with unfavorable patterns of spread. The beneficial effect of whole-brain radiotherapy is not necessarily limited to symptom palliation.

## Introduction

Breast cancer is often diagnosed in early stages and has the potential to recur with distant metastases after variable length of follow-up, despite standard locoregional and systemic therapy [1]. Synchronous distant metastases at first cancer diagnosis (de novo stage IV disease) are less common. The presence of liver metastases may indicate an unfavorable prognosis, compared to, for example, bone metastases alone [2]. However, both patterns of disease, response to treatment, and survival are highly variable [3]. Breast cancer biology, e.g., expression of estrogen (ER), progesterone (PR) and HER2 receptors, profoundly influences the choice of systemic therapy and survival in patients with stage IV disease [4]. Patients with HER2 overexpressing metastatic breast cancer are at increased risk for development of brain metastases [5]. Median survival after diagnosis of brain metastases in different studies is in the range of 6-18 months, as reviewed in [5-6]. Long-term survival is possible, especially in younger patients with good performance status. The present case report illustrates several important issues related to metastatic HER2 overexpressing breast cancer.

## Case presentation

**Figure 1:**
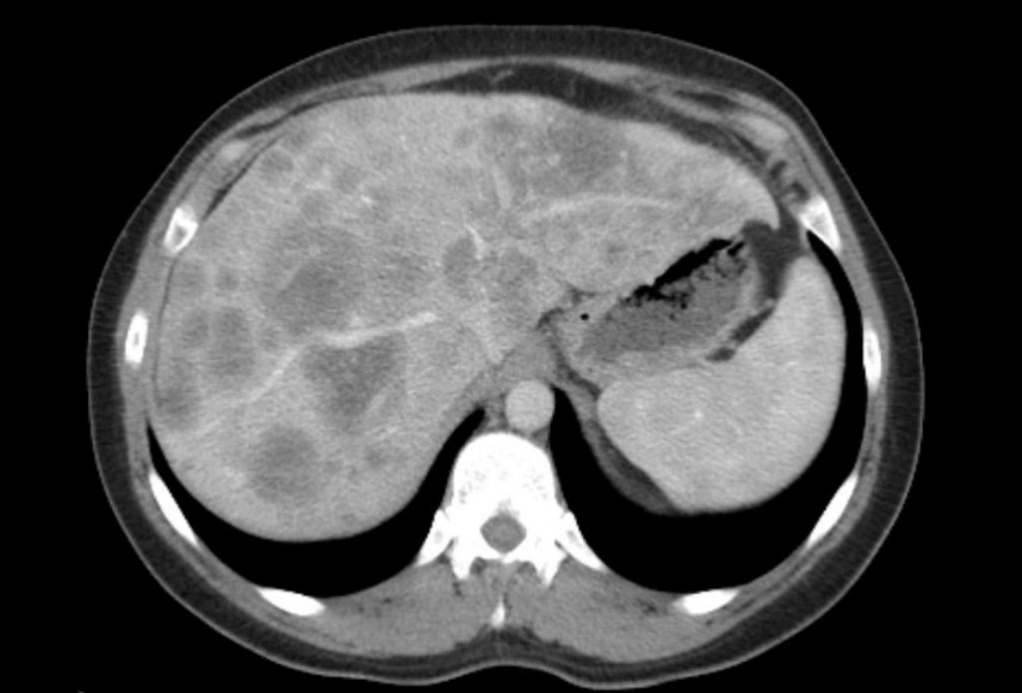
Liver metastases (computed tomography)

A 35-year-old female Caucasian patient palpated a mass in her right breast in February 2011. Staging examinations revealed a T1c primary tumor, axillary lymph node metastases, and multiple liver metastases up to 45 mm throughout the liver (Figure 1).

**Figure 2:**
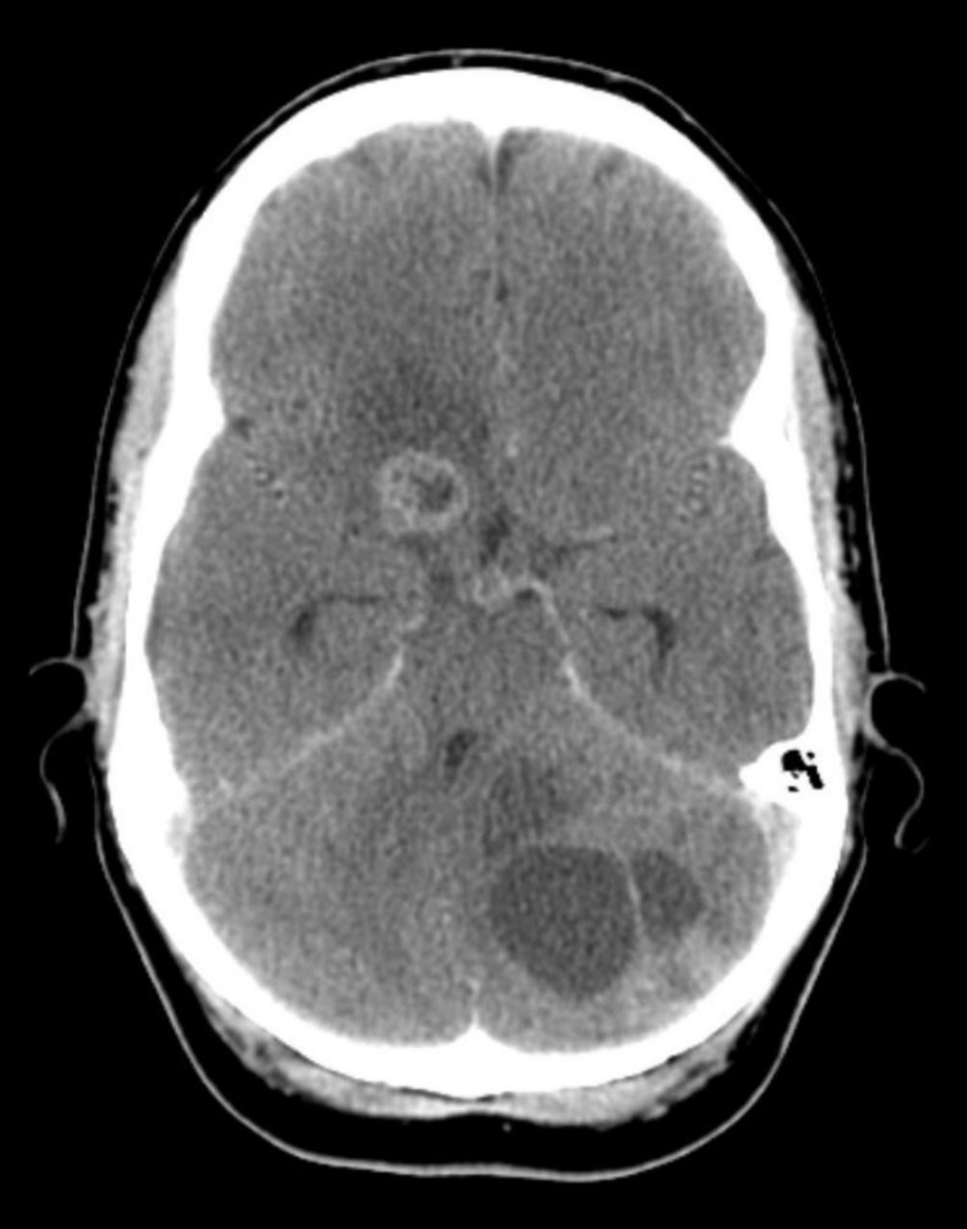
Brain metastases (computed tomography)

Histology confirmed poorly differentiated invasive ductal carcinoma, ER and PR negative, HER2 positive, and the patient was negative for germline BRCA 1 and 2 mutations. The serum tumor marker CA 15-3 was elevated to 620 kIE/l. Emerging biomarkers such as circulating tumor cells were not assessed. The patient was otherwise healthy and in excellent performance status. First-line systemic treatment with weekly docetaxel and trastuzumab was initiated in March. Treatment with docetaxel was stopped in August because of peripheral neuropathy. All sites of disease were in complete clinical remission at that time. CA 15-3 was no longer elevated (10 kIE/l). The patient continued on trastuzumab, now every three weeks. In September 2012, after increasing problems with headaches, multiple supra- and infratentorial parenchymal brain metastases were diagnosed. Computed tomography revealed six lesions with a maximum diameter of 40 mm of solid or cystic appearance (Figure 2).

No other active sites of disease were present. CA 15-3 was still normal. The patient's performance status was excellent. Whole-brain radiotherapy was initiated (10 fractions of 3 Gy). Trastuzumab was continued. When evaluated for delayed radiosurgery to residual brain metastases, all lesions had resolved completely. The clinical course was unremarkable until May 2016 when mammography showed recurrent tumor in her right breast (after 86 courses of trastuzumab), again without accompanying increase in CA 15-3. No lymph node or other metastases were detected. A mastectomy was performed and the histology was identical to the initial findings from 2011. Postoperatively, systemic therapy was changed to paclitaxel, trastuzumab, and pertuzumab. The patient is currently still on active treatment with this regime (last follow-up March 06, 2017). No serious cardio- or neurotoxicity has occurred.

## Discussion

The patient presented in this report was diagnosed with de novo stage IV HER2-positive breast cancer with distant metastases in an unfavorable organ site, i.e. liver. She responded completely to systemic therapy with a taxane and trastuzumab. In this situation, controversy exists around the usefulness of additional locoregional therapy [1, 3]. We opted against local treatment of the breast and axillary lymph nodes because the disease extent in the liver was much larger. From today's point of view, locoregional therapy would have been beneficial, because the in-breast recurrence could likely have been prevented. As repeatedly reported in the literature, HER2-positive breast cancer is often associated with the development of brain metastases [4-5]. Our patient was not screened with regular imaging, consistent with national guidelines and clinical practice. She was diagnosed after the development of clinical symptoms, and based on the number and size of the lesions, she was considered not a good candidate for upfront surgery or radiosurgery. The concept of whole-brain radiotherapy and delayed radiosurgery to residual disease was favored. Unexpectedly, imaging revealed complete resolution of the brain metastases. Therefore, the patient continued on trastuzumab alone. Whole-brain radiotherapy with 10 fractions of 3 Gy is rarely associated with long-term local control. Additional systemic agents might cross the blood-brain barrier and contribute to better response [6]. Fortunately, this young, otherwise healthy and active patient is currently alive more than four years after radiotherapy. In her situation, optimal brain control was a crucial prerequisite for long-term survival. Even if she has not experienced clinically obvious neurotoxicity, today's recommendation would probably include hippocampus-sparing rather than standard whole-brain radiotherapy. Given that the primary tumor recurred after more than five years, the patient is still at risk of failure in the brain, liver, and other distant sites. However, given the excellent response to a taxane and trastuzumab, her current treatment that also includes pertuzumab is hoped to prevent further recurrence. Identification of biomarkers other than HER2 that might be associated with exceptional responses like the one observed here would be of great interest. Personalized and timely therapy is crucial in the context of metastatic breast cancer, a biologically heterogeneous disease with increasing options for systemic treatment, resulting in a growing proportion of long-term survivors.

## Conclusions

Even patients with liver and brain metastases do not share a uniformly fatal course of disease with typical survival of two years or less. As illustrated here, both HER2-directed systemic therapy and radiotherapy are sometimes able to cause long-lasting, successful remission. Findings derived from uncontrolled small studies or case reports cannot prove the benefits of a certain type of treatment. However, the present case confirms observations made in previous prospective studies. Prevention of brain metastases remains of high importance in the setting of HER2-positive disease. Exceptional responses to standard, guideline conform treatment confirm that further efforts to predict individual chemo- and radiosensitivity are warranted.
